# Novel Biomarkers and Treatments of Cardiac Diseases

**DOI:** 10.1155/2016/1315627

**Published:** 2016-02-17

**Authors:** Hua Zhu, Renzhi Han, Dayue Darrel Duan

**Affiliations:** ^1^Department of Surgery, Davis Heart and Lung Research Institute, The Ohio State University, Wexner Medical Center, Columbus, OH 43210, USA; ^2^Laboratory of Cardiovascular Phenomics, Center for Cardiovascular Research, Department of Pharmacology, and Center for Molecular Medicine, University of Nevada School of Medicine, Reno, NV 89557-0318, USA

Cardiac diseases are mainly caused by malfunction of or injuries to the hearts. Although significant advances have been made during the past decades to improve the successful rate of treatments of these diseases, they still remain the top leading cause of morbidity and mortality in the world. Currently, cardiac diseases are defined according to the traditional system- or organ-based classification and the identification of diagnostic and therapeutic biomarkers has been focused on the heart. Therefore, the “golden standard” biomarkers are mainly cardiac muscle-related. For example, the conventional troponin (cTn) has been widely utilized for the diagnosis of acute myocardial infarction in the clinics. However, multiple limitations exist with clinical applications of these types of biomarkers. The ELISA based cTn detection assay is time consuming, and the dynamics of the biomarkers are not sensitive enough to represent the development of the diseases. Clearly, there is an urgent need for identifying sensitive, specific biomarkers of different types of cardiac diseases and development of new therapies for this unmet medical need. In this special issue, we have assembled a series of articles of reviews, perspectives, and original contributions from experts in current research of novel biomarkers and treatments of cardiac diseases in both basic research and clinical practice.

In the effort to develop more sensitive cardiac specific troponin-based diagnosis for heart diseases, the highly sensitive troponin T (hsTnT) was found to offer an excellent diagnostic performance due to its high sensitivity and negative predictive values compared with conventional troponin (cTn) test. In this special issue, the potential clinical use of hsTnT for diagnosing perioperative myocardial infarction (PMI) was discussed. In addition, it was discovered that hsTnT combined with advanced oxidation protein products (AOPPs) could be useful for monitoring myocardial function of cirrhotic patients with chronic hepatitis C virus infection.

Other than the circulating troponins, many other proteins have been tested recently as biomarkers for cardiac diseases. For example, caveolin-1, a membrane protein, has been found in blood circulation and might serve as a novel biomarker for idiopathic pulmonary artery hypertension. Serum level of pentraxin-3, a member of pentraxin family, is a long-term independent predictor of prognosis of patients with chronic heart failure.

In addition to circulating proteins, other circulating molecules have also been discovered as biomarkers in many disease settings. MicroRNAs are small RNAs with ~22 nucleotides in length that can regulate specific messenger RNAs (mRNAs). They present in both tissues and blood circulation and can be used as biomarkers of cardiac diseases [1]. In one research article of this special issue, N. Li et al. found that miR-1183 and miR-1299 in both tissue and plasma can serve as biomarkers for rheumatic heart disease (RHD). Recently, microRNAs have been found in extracellular vesicles (EVs) and can be altered in association with cardiovascular diseases.

Some other molecules have also been investigated for the value of biomarkers in cardiac diseases. For example, alteration of preoperative and postoperative plasmatic endogenous ouabain (EO) has been linked to patients with higher risk of morbidity and mortality after cardiac surgery. The differential expressed genes (DEGs) in human epicardial adipose tissue (EAT) can serve as biomarkers as well as therapeutic targets for treatment of cardiovascular diseases. A research article in this special issue also demonstrated that activation of endocannabinoid system, as evidenced by elevation of cannabinoid receptors, infiltration of leukocytes and mononuclear cells, is associated with persistent inflammation in human aortic aneurysm. As a novel therapeutic strategy for the treatment of cardiac diseases,* Withania somnifera* leaf extract, one of the most valuable herbs in the traditional Indian systems of medicine, can effectively treat isoproterenol-induced oxidative damage in rat myocardium.

Taken together, the studies assembled in this special issue represent several important paradigm shifts in the area of biomarkers for the diagnosis, prognosis, and treatment of cardiac diseases. The identification of biomarkers for the diagnosis and treatment of cardiac diseases should not be limited to the organ- or tissue-specific molecules (proteins or nucleotides). The systematical nature of pathogenesis and pathophysiology of cardiac diseases requires broader vision and strategies outside the organ- or system-based box [2]. The fast advance in the application of genome-wide association study (GWAS) [3], phenome-wide association study (PheWAS) [4, 5], and epigenome-wide association study (EWAS) [6], as well as big-data technology provides more powerful platforms and paradigms for the new definition of cardiac diseases, which will be based on not only genetic makeups and molecular variations but also environmental impacts. The identification of systematically integrated biomarkers is certainly a crucial step towards precision medicine [7] to optimize diagnosis and treatment of cardiac diseases that takes into account individual differences in molecular makeups, life styles, and environmental impacts.
